# Validation of an integrated metagenomic pipeline combining optimized wet-lab processing and tiered reporting for CSF pathogen detection

**DOI:** 10.1128/spectrum.03666-25

**Published:** 2026-06-29

**Authors:** Alec Victorsen, Todd P. Knutson, Lucas Bolender, Sabrina Jung, Patricia Ferrieri, Bharat Thyagarajan, Evann E. Hilt

**Affiliations:** 1 Department of Laboratory Medicine and Pathology, University of Minnesota 5635, Minneapolis, Minnesota, USA; 2 Minnesota Supercomputing Institute, University of Minnesota 5635, Minneapolis, USA; University of Saskatchewan, Saskaton, Canada

**Keywords:** infectious disease diagnostics, clinical genomics, microbiology

## Abstract

Metagenomic next-generation sequencing (mNGS) in the infectious disease diagnostic space has been gaining traction and is popular for aiding in the diagnosis of central nervous system infections. However, many challenges and obstacles remain in making this technology a gold standard for infectious disease diagnostic testing. One major challenge is being able to distinguish between the clinically relevant organisms from background contamination. We performed a validation study for mNGS on cerebrospinal fluid (CSF) that utilized positive clinical samples and contrived samples that incorporated a bioinformatics pipeline that can better distinguish between background contamination and clinically relevant organisms and used a three-tiered reporting algorithm meant to decrease the inherent subjectivity that comes with interpreting and reporting data from clinical metagenomic sequencing. The validation of this assay and category-based reporting pipeline revealed an overall concordance of 91.8%, with a sensitivity of 100% and a specificity of 72.4%. In addition, we improved the detection of clinically relevant RNA viruses to almost 100% in the CSF by modifying the wet lab processing of the sample. This bioinformatics pipeline with a category-based reporting algorithm will provide more confidence in reporting microorganisms detected with this technology, mNGS, and improving patient care.

**IMPORTANCE:**

Metagenomic next-generation sequencing (mNGS) can offer a broad, unbiased approach for the detection of infectious pathogens and has shown promise in diagnosing central nervous system infections. Despite its potential, clinical implementation remains limited by challenges in distinguishing clinically relevant organisms from background contamination. This study validated an mNGS assay for cerebrospinal fluid that incorporates an optimized bioinformatics pipeline with a three-tiered reporting algorithm designed to reduce subjectivity and enhance diagnostic confidence. The assay also has improved detection of clinically relevant RNA viruses through modified wet-lab processing. These findings support the clinical utility of a structured, category-based reporting approach for mNGS, advancing its reliability as a diagnostic tool in infectious disease testing.

## INTRODUCTION

Central nervous system (CNS) infections, including meningitis, encephalitis, and myelitis, are a significant cause of morbidity and mortality worldwide (1). The use of molecular diagnostics, specifically multiplex PCR assays, is helpful for diagnosis in some of these CNS infections, but these panels only target a small number of potential pathogens that are known to cause disease (2). Despite extensive testing, up to 30% of CNS infections do not have a known etiology (3–6), creating substantial challenges for clinical management.

Metagenomic next-generation sequencing (mNGS) offers a hypothesis-free approach capable of detecting a broad range of potential pathogens in a single diagnostic assay (7–11). A common challenge faced by laboratories is the ability to differentiate between the clinically relevant pathogens and clinically irrelevant background contaminants (9, 12). Microbial contamination is widespread, even in the reagents used to extract and purify nucleic acids (13, 14). Establishing a bioinformatic threshold to distinguish true microbial pathogens from background contaminants is a commonly used method to address this problem (7, 9). Furthermore, current mNGS workflows are limited by the labor-intensive interpretation required to identify clinically relevant pathogens, as well as by a poor sensitivity for the detection of RNA viruses.

Here, we present an mNGS assay for cerebrospinal fluid (CSF) that addresses the limitations of the current mNGS methods. This includes the development of a bioinformatics pipeline designed to report pathogens based on a tiered threshold system that maximizes the ability to objectively identify the most abundant potential microbes in the CSF samples and a method optimized for the detection of RNA viruses. In addition, we present critical metrics to establish and monitor assay integrity when validating mNGS for clinical testing.

## MATERIALS AND METHODS

### Validation studies

This mNGS assay on CSF was tested for the following metrics of a laboratory-developed test: accuracy with a discordant analysis, precision, analytic sensitivity as a limit of detection (LOD), analytic specificity with cross-reactivity and interference, and the establishment of both external and internal controls. The types of samples used for the validation studies are described below. This validation study for the mNGS assay on CSF included a comparison to reference methods where clinical samples were positive by conventional methods. In addition, several recovery experiments (including limit of detection) were performed, where contrived samples were made to test targets that were not available in real-time clinical samples.

### Sample selection

#### Clinical samples (52 samples)

Excess CSF samples, obtained via a lumbar puncture and submitted to the Infectious Diseases Diagnostic Laboratory (IDDL) at M Health Fairview, Minneapolis, MN, for microbiologic diagnostic testing, were used in this study. All positive clinical samples were either positive by the BioFire Meningitis/Encephalitis (M/E) PCR Panel (bioMérieux, Marcy-l’Étoile, France) or from conventional bacterial or fungal cultures. All the negative clinical samples had been submitted for routine testing (M/E panel and culture) and were negative by both methods. After testing, samples were frozen and stored at −80°C with minimal freeze-thaws, and any transfer was performed on ice. All samples were residual and de-identified clinical samples that were used exclusively for clinical test validation; therefore, IRB approval was not required.

#### Contrived samples (13 samples)

In brief, CSF samples that were negative by both PCR and culture were combined to create a pooled negative sample for use as the matrix for these contrived samples. The following bacterial and fungal organisms were grown overnight in CO_2_ atmospheric conditions: *Listeria monocytogenes* (ATCC 7646)*, Cutibacterium acnes* (patient isolate)*, Streptococcus agalactiae* (ATCC 12386)*, Neisseria meningitidis* (ATCC 13090)*, Escherichia coli* (ATCC 25922)*, Haemophilus influenzae* (ATCC 10211), *Candida albicans* (ATCC 10231), and *Cryptococcus neoformans* (ATCC 13690). A 0.5 McFarland was made in saline, creating a 1.5 × 10^8^ colony-forming unit (CFU)/mL concentration of each bacterium and a 10^6^ CFU/mL concentration for each fungus. For LOD studies, each bacterial organism was added to negative CSF and diluted down to a concentration of 100 CFU/mL. Each fungal organism was added to negative CSF and diluted down to a concentration of 1 CFU/mL. These contrived samples were used as either contrived positive CSF samples to further test the accuracy of the assay for *L. monocytogenes, C. acnes, S. agalactiae, N. meningitidis, E. coli, H. influenzae*, *C. albicans,* and *C. neoformans,* and for limit of detection studies. For viral contrived samples, pure quantitative genetic content of the viral organisms (Varicella Zoster Virus [ATCC VR-1367DQ] and Enterovirus [ATCC VR-1826DQ]) was purchased from ATCC. For the viral LOD studies, each virus had a starting concentration of ≥1 × 10^6^ copies/µL. Serial dilutions of the ATCC stock solutions were prepared, starting with a 1:5 dilution, followed by five serial 1:10 dilutions. For each dilution, 1 µL was added to total nucleic acid (TNA) extracts from negative CSF samples.

#### Control samples

External negative controls were 600 µL of nuclease-free water. A positive external control sample was also generated by combining 5 ng of ZymoBIOMICS Microbial Community DNA Standard (Zymo) with 1 µL ATCC Virome Nucleic Acid Mix in 600 µL water. External negative and positive controls were run in tandem with each batch.

### Total nucleic acid purification

**Fig 1:**
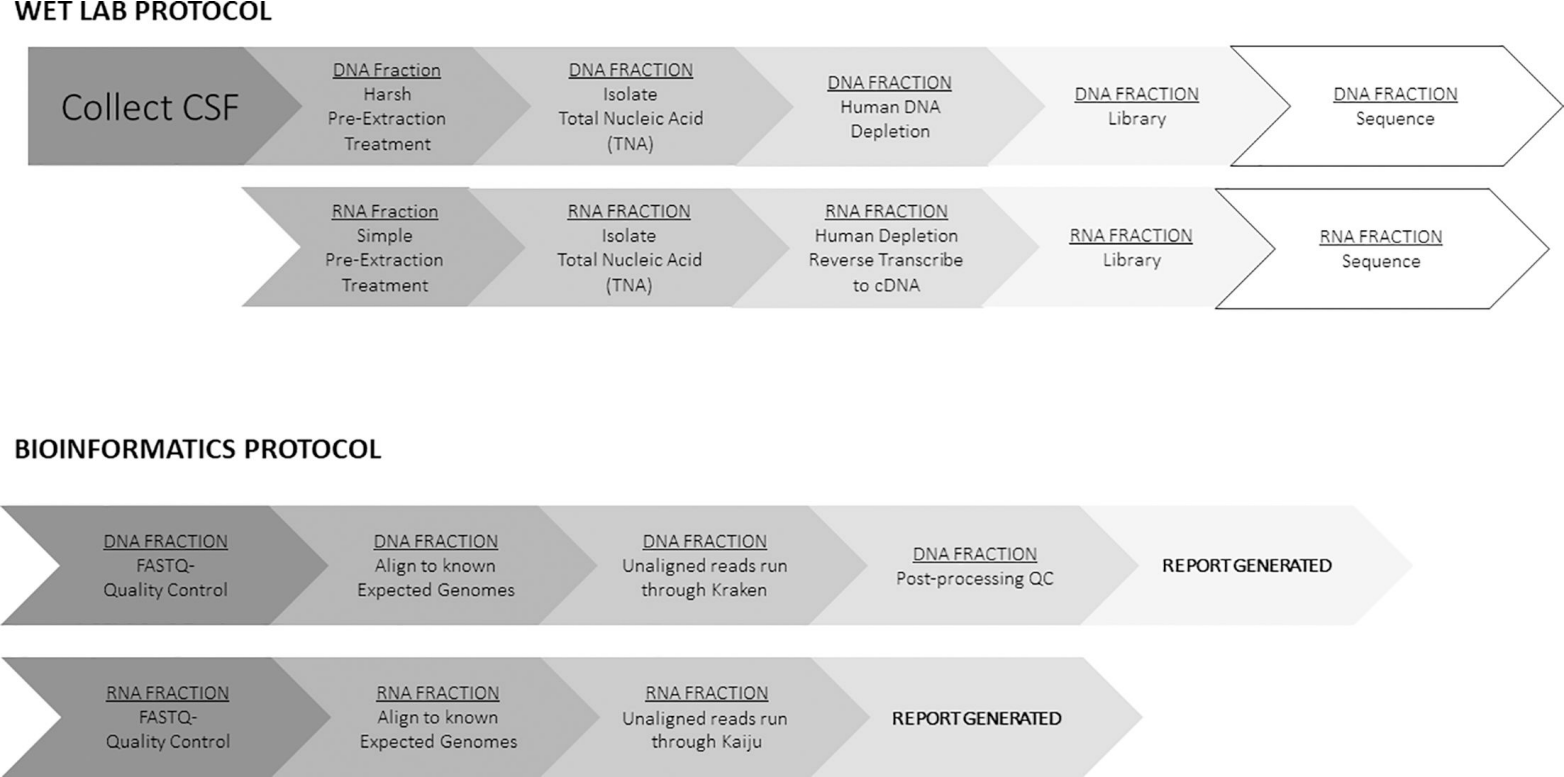
Overview of mNGS assay protocols**.** The wet-lab protocol involved splitting the initial CSF sample into separate DNA and RNA fractions and keeping those fractions separated during the duration of the wet-lab protocol, sequencing, and bioinformatics analysis.

An overview of the wet-lab protocol adapted from Miller et al. is detailed in Fig. 1 with some modifications (7). CSF was split into two fractions for separate DNA and RNA wet-lab protocols. The DNA fraction was processed using a pre-extraction protocol that included a bead beating step to lyse the cell wall in gram-positive bacteria, whereas the bead beating step was excluded in the RNA fraction. In short, the DNA fraction was purified by combining 400 µL of CSF with 200 µL of nuclease-free water to a matrix B tube (MP bio) and lysed on an MP bio FastPrep-24 twice at 6 m/s for 30 s with a 5 min incubation on ice in between rounds. The RNA fraction (200 µL of CSF) did not undergo this lysing step, a modification from Miller et al. (7). Both DNA and RNA fractions were centrifuged at 2,000 × *g* for 5 min. For internal controls, we added 83.2 pg of MS2 phage RNA and 1 ng of Lambda phage DNA to each sample. Both the DNA and RNA fractions had TNA extracted and purified using the Promega Maxwell system and the Total Nucleic Acid Purification Kit (Promega, Madison, WI, USA). The TNA for the DNA and RNA fractions was eluted in 50 µL water.

### DNA fraction—human depletion step

The concentration of the TNA purification for the DNA fraction was measured on a Qubit with the dsDNA HS Assay Kit. Samples were diluted if necessary to <10 ng/µL, and 50 µL was processed using the NEBNext Microbiome Enrichment Kit (New England Biolabs [NEB], Ipswich, MA, USA) with 50 µL of magnetic beads. The unbound supernatant was removed and treated with 1 µL RNase A (NEB) for 15 min at 25°C and then purified using the Zymo DNA Clean and Concentrator Kit with 16 µg/mL linear acrylamide added to the binding buffer. Samples were eluted in 30 µL water.

### RNA fraction—human depletion step and conversion to cDNA

The RNA fraction of the TNA was digested in 100 µL reactions using 5 µL Turbo DNase (ThermoFisher, Waltham, MA, USA), 2 µL Baseline-ZERO DNase (Lucigen), and 2 µL SUPERase-IN (ThermoFisher, Waltham, MA, USA) in 1× Turbo DNase buffer at 37°C for 30 min. Digests were purified using the Zymo RNA Clean and Concentrator MagBead Kit and eluted in 15 µL water. To the eluted RNA, 1 µL of SUPERase-IN was added along with 1.25 mM dNTPs and 50 µM random hexamers in a volume of 25 µL. This mixture was incubated at 65°C for 5 min. The RNA was then converted into cDNA by adding 1 µL of SuperScript III Reverse Transcriptase (ThermoFisher, Waltham, MA, USA) in First-strand buffer and 10 mM DTT in 50 µL and incubated at 25°C for 5 min, 50°C for 50 min, and 95°C for 15 min. After chilling the samples, the second strand was synthesized by adding 0.5 µL Sequenase (ThermoFisher, Waltham, MA, USA) in a reaction buffer supplemented with an additional 1 mM MgCl_2_ and incubated at 37°C for 15 min. Residual RNA was removed by adding 1 µL of RNase A (NEB) and digesting for 15 min at room temperature. The cDNA was purified using the Zymo DNA Clean and Concentrator Kit with 16 µg/mL linear acrylamide added to the binding buffer. Samples were eluted in 30 µL water.

### Library preparation and sequencing

Illumina libraries on both DNA and RNA fractions were built using the Illumina DNA Prep (M) Tagmentation kits with the IDT for Illumina DNA/RNA UD indexes Set B, Tagmentation indexes (Illumina, San Diego, CA, USA). Final libraries were eluted in 12 µL water rather than resuspention buffer (RSB). A second round of PCR amplification of the Illumina libraries was performed with Phusion polymerase (ThermoFisher, Waltham, MA, USA) by combining 5 µL Illumina library sample, 0.2 mM dNTPs, and 0.5 µM P5/P7 Primer mix (IDT) in HF buffer in 20 µL reactions. Amplified libraries were purified with AMPure XP beads (Beckman Coulter) and resuspended in 20 µL RSB. Library concentrations were quantified on the Qubit with the dsDNA HS Assay Kit. Libraries were pooled and sequenced on an Illumina NextSeq 2000 with the 300 cycle P2 flow cells. A minimum of 10 M PE reads was required for each sample.

### Bioinformatics pipeline

An overview of the bioinformatics pipeline is detailed in Fig. 1. Data were processed at the University of Minnesota Supercomputing Institute (MSI). A custom Kaiju database was compiled by assembling all riboviria genomic sequences in NCBI (15). A custom Kraken2 database was compiled using all known genomes and scaffolds in NCBI for archaea, bacteria, viruses, fungi, and protozoa, as well as the human genome reference consortia assembly (16, 17).

The quality of FASTQ files was assessed several times during processing using FastQC (on raw, trimmed, and unique) (18). Adapters and low-quality sequences were removed using Trimmomatic (v0.36) (19). A concatenated list of potential adapters was generated from those supplied with Trimmomatic, and an additional list of options listed in Table 1 was used. Sequences shorter than 50 base pairs (bp), with more than two *N*s, dust scores <0.93, or mean quality scores <30, were removed using PRINSEQ++ (v1.2.4) (20).

**TABLE 1:** List of additional options in Trimmomatic (v0.36)

Parameter	Value
seedMismatches	2
minPalindromLikelyhood	30
minSequenceLikelyhood	10
minPrefix	1
palindromKeepBoth	True
MAXINFO	100:0.8
SLIDINGWINDOW	6:20
LEADING	6
TRAILING	6

The high-quality reads were aligned using BWA to the hg38 human genome, human transcriptome if generated from an RNA sample, Lambda phage, and MS2 phage genomes. The remaining paired reads were filtered with PRINSEQ++ to remove duplicate sequences (-derep 235). For DNA-derived samples, Kraken2 was used to assign reads to taxa. Graphs were generated using Krona (v2.7) (21). For RNA-derived data, unique reads were assigned to taxa with Kaiju (v1.6.0) using a loose method (match length > 11 with three mismatches) or a strict method (match length > 25 with five mismatches). The strict method was used to generate reports.

**Fig 2:**
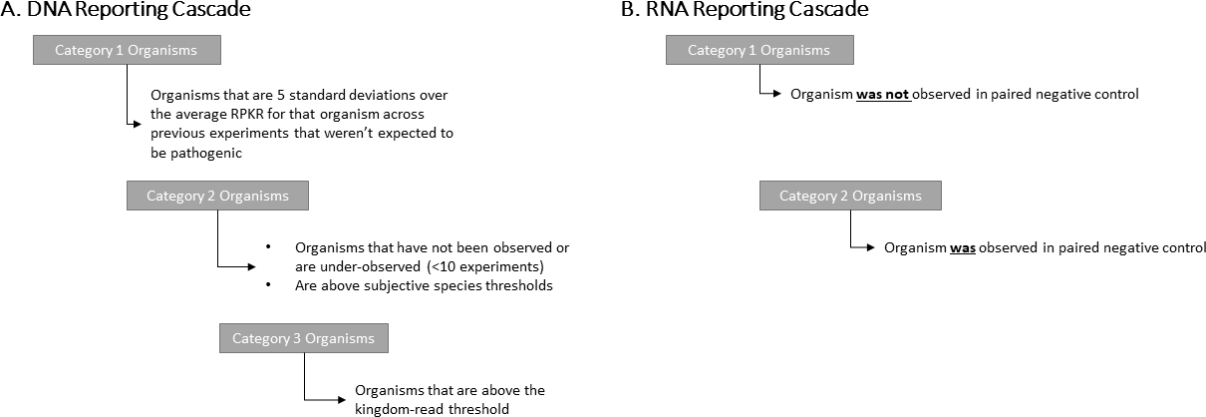
Reporting cascade for mNGS assay. (**A**) DNA reporting cascade**:** the first category utilizes a statistical method of reporting organisms that are detected at RPKR values that are 5 standard deviations over the average RPKR for that organism across previous sequencing runs presumed to be negative for that organism. The second category is composed of organisms that are under-observed in previous runs (*n* < 10) and are above the kingdom threshold. The third category of DNA reporting cascade contains organisms that are above the kingdom-read threshold. (**B**) RNA reporting cascade: the two categories are separated by whether the ribovirus was not observed or was observed in the paired negative control.

Consolidated reports were generated for each sample type to include relevant read counts (total, Human, Lambda phage, MS2 phage, and unique reads) (Fig. S1 and S2). Kaiju and Kraken outputs were parsed and added to these reports. Species-assigned read counts from Kraken2 were only reported if they were above empirically determined minimum kingdom thresholds below which any reads were likely to be anomalous (Archaea: 10, Bacteria: 200, Eukaryota: 100, and Viruses: 3). To compensate for the differences in sequencing depth between samples, we began using a ratio of “reads per thousand unique reads (RPKR).” The RPKR ratios for species above the kingdom thresholds were calculated, and the RPKR thresholds were used to develop a “category-based” system of reporting for the DNA organisms (Fig. 2, DNA reporting cascade). The first category utilizes a statistical method of reporting organisms that are detected at RPKR values that are 5 standard deviations over the average RPKR for that organism across previous sequencing runs presumed to be negative for that organism. This equates to a roughly 1 in 5 million chance of a false positive if normally distributed. The second category is composed of organisms that are under-observed in previous runs (*n* < 10) and are above an empirical threshold set for known clinically relevant organisms. The third category of DNA reporting cascade reports all remaining organisms that are above the kingdom-read threshold. This third category allows all the information of what is sequenced to be seen and reviewed. Read counts assigned to RNA viruses by Kaiju were separated into two categories based on whether or not the virus was observed in the paired negative control (Fig. 2, RNA reporting cascade).

## RESULTS

### Overall concordance and validation results

The entire assay (wet-lab protocol and bioinformatics protocol) underwent an analytical validation study for accuracy, precision, analytical sensitivity, analytical specificity, and establishment of controls (Table 2).

**TABLE 2:** Summary of validation results for mNGS assay^*a*^

Study	Results	Acceptance criteria	Criteria met? Yes/No
Adjusted concordance	Adjusted concordance: 91.9%	≥90%	Yes
	Sensitivity: 100%		
	Specificity: 72.4%		
	Positive predictive value: 89.7%		
	Negative predictive value: 100%		
Precision: within run	100%	100%	Yes
Precision: between run	100%	100%	Yes
Analytical sensitivity Limit of detection	Bacterial: 100 CFU/mL	N/A	Yes
	Fungal: 1,000 CFU/mL		
	DNA virus: 10 viral copies/µL		
	RNA virus: 10 viral copies/µL		
Analytical specificity Cross-reactivity Interference	Established	N/A	Yes
Establish controls	Established	N/A	Yes

^*a*^ N/A, no predefined acceptance criterion was established for this metric during validation.

A total of 86 individual CSF samples were tested in this validation, including 21 negative samples and 65 positive samples (both clinical and contrived). Several of the positive samples were run in duplicate or triplicate to complete the precision portion of the validation, so a total of 99 tests were run (Table S1). The resulting validation, after an adjusted concordance analysis (Supplemental material—Adjusted Concordance Analysis), yielded a 100% sensitivity and 72.4% specificity with an overall concordance of 91.9% compared to either the original test result or the known spiked organism. This mNGS assay validation had the focus of detection of five classes of organisms: bacteria (gram-positive and gram-negative), fungi, and viruses (DNA and RNA). When looking deeper into the sensitivity and specificity into each organism class (Table 3), the driver of the overall low specificity was the detection of RNA viruses that had a sensitivity of 100% and a specificity of 84%.

**TABLE 3:** Breakdown of the category-based detection of organism class^*a*^

	Sensitivity (%)/specificity (%)	DNA category 1 detection	DNA category 2 detection	DNA category 3 detection	Not detected
Gram-positive bacteria (total = 16)	100/95.5	13	–	3	–
Gram-negative bacteria (total = 10)	100/100	10	–	–	–
Fungi (total = 9)	100/95.5	6	–	2	1
DNA virus (total = 24)	100/91.3	–	22	–	2

	Sensitivity/specificity	RNA category 1 detection	RNA category 2 detection		
RNA virus (total = 18)	100/84	13	1		4

^*a*^ “–” indicates that no organism was detected in the corresponding category.

The precision within and between runs of samples was 100% detection of the organism of interest. The LOD for both gram-positive and gram-negative bacteria was down to 100 CFU/mL. For fungal organisms, the mNGS assay was only able to detect down to around 1,000 CFU/mL, but this could be due to loss during the methylated DNA depletion step. For both DNA and RNA viruses, the mNGS assay was able to detect down to 10 viral copies/µL (Table 2).

### Category-based reporting

During the development of this assay, the method used to determine true reads from background evolved. After a few sequencing runs, the large number of organisms present at extremely low abundance was evident. As a result, we implemented kingdom-level thresholds that seemed reasonable and removed many of the organisms whose total reads amounted to fewer than 0.005% of the non-human usable reads (Archaea: 10, Bacteria: 200, Eukaryota: 100, and Viruses: 3). In our positive samples, the number of on-target reads still represented a small fraction of the unique reads while the “kitome” dominated. The “kitome” is known as DNA contamination from the extraction and reagent kits (22). Therefore, to normalize background-read levels between experiments, we implemented the RPKR value as a ratio between the taxa read counts per thousand unique reads.

After implementing these limits and metrics, we continued to observe low levels of clinically relevant bacteria in samples that were purported to be negative, which we attributed to the “kitome.” For reference, these false positives varied by species but constituted approximately fewer than 0.01% of unique reads for *S. pneumoniae* and 0.09% of unique reads for *E. coli*. In contrast, the true positive percentages for these species were 0.2%–40.3% and 2.5%–22.5%, respectively. Since the RPKR levels in true positive samples were much higher, we implemented species-level thresholds for several common organisms (see Fig. S1). However, rather than continuing to rely on empirical thresholds, we improved on this method once we had sequenced approximately 75 samples and systematically calculated RPKRs for all observed non-true positive organisms across these samples. Using the RPKR distributions, organisms that are at significantly higher than normal levels within a sample can be flagged. Each of these methods continues to be used to hierarchically grade all observed organisms above the kingdom levels in our categorical reports to mitigate any false negative call. This stands in stark contrast to the RNA reports, where due to restricting the Kaiju database to only Riboviria, raw reads were enough to highlight putative pathogens. We chose not to implement any cutoffs due to the inherent nature of RNA viruses being difficult to detect, and any detection of these highly putative pathogens would initiate a discussion among the clinical team evaluating and treating the patient.

The bioinformatics pipeline and report generated for the mNGS assay utilized a separate category-based reporting system for DNA and RNA samples (Fig. 2). For the DNA cascade, Category 1 organisms were determined based on a statistical method utilizing the RPKR values. For example, if *Staphylococcus aureus* was reported in Category 1, then *S. aureus* was seen at an RPKR value that was 5 standard deviations over the average RPKR value of *S. aureus* across all previously sequenced *S. aureus* negative samples. This would indicate that *S. aureus* is a primary organism seen in the sample and should be reported as present in that sample. Category 2 in the DNA cascade is used to help capture any unique organisms that may be of clinical significance but are not frequently observed in samples. Finally, Category 3 in the DNA cascade is meant to show the remaining organisms detected that do not meet the other set thresholds. Four classes of organisms fell into the DNA reporting cascade: gram-positive bacteria, gram-negative bacteria, fungi, and DNA viruses. In the DNA samples for both bacterial classes and fungi, most of the organisms were detected in Category 1 (Table 3). Specifically, in the gram-positive and fungi samples, a percentage of samples (19% and 25%, respectively) were only detected in Category 3. All the CSF samples positive for DNA viruses were detected in Category 2. These results indicated that these DNA viruses were unique and not frequently observed in previously sequenced samples.

In the RNA virus samples, most (13 of 14) were detected in Category 1, which detected RNA viruses that were not seen in the paired negative CSF sample (Table 3).

The category-based reporting system on CSF minimizes the ambiguity and subjectivity that come with reporting pathogens from metagenomic sequencing. Most of our samples (55%, 42/77) had the organisms detected in the first category, regardless of whether it was a DNA or RNA sample (Table 3). If we were to remove the class of DNA viruses, this would change the percentage to 76% (40/55) detection in Category 1.

### Case examples of reporting clinically relevant organisms that were not reported previously

The advantage of mNGS technology is the ability to identify all potential pathogens in a sample instead of having to order an array of tests/assays to obtain a comprehensive workup. There were two samples where the category-based reporting helped to flag other potential important pathogens (Table 4). Both samples were initially positive for Enterovirus, and this organism was detected in our RNA cascade pipeline in Category 1. In the DNA cascade pipeline, both samples had clinically relevant pathogens detected (Table 4). In sample Study #53, *Klebsiella pneumoniae* was detected in DNA Category 1, which would have prompted the laboratory team to initially report out this pathogen. Interestingly, in sample Study #18, *Listeria monocytogenes* was detected in Category 3, which may prompt providers to question whether it was valid. However, the RPKR threshold for *L. monocytogenes* RPKR value in Category 3 was 1.3695, and an RPKR result of 7.9314 was several folds higher than that value, which would have prompted the team to report out *L. monocytogenes* initially.

**TABLE 4:** CSF samples with detection of additional potential pathogens

Sample ID	Original PCR/Culture result	mNGS result					
		RNA ID	Normalized reads to MS2	Category detected	DNA ID	Normalized RPKR to Lambda	Category detected
Study #18	Enterovirus	Enterovirus B	18 reads	1	*Listeria monocytogenes*	7.9314	3
Study #53	Enterovirus	Enterovirus B	1,113 reads	1	*Klebsiella pneumoniae*	1.2164	1

### Increased sensitivity for detection of RNA viruses

Initial LOD studies for the RNA virus, Enterovirus, revealed a very high LOD, which suggested that the mNGS assay was not optimal for the detection of RNA viruses (Table 5). This correlated with the initial high discordance observed with five of the nine RNA-positive CSF samples being negative by mNGS. This was not surprising given the unstable nature of RNA, but an investigation into the sequencing output showed that the MS2 RNA internal control phage was not being amplified, as well. We hypothesized that the initial pre-extraction treatment method was too harsh for robust RNA virus detection, so we modified this part of the wet-lab protocol to improve the detection of RNA viruses. In brief, for the RNA fraction, 200 µL of CSF was combined with the Lambda and MS2 controls and purified on the Maxwell without prior bead beating steps and eluted in 50 µL of water. Furthermore, we felt that SDS from RNAseZap was contaminating the RT-PCR reaction, thus hindering detection; therefore, it was used less gratuitously.

**TABLE 5:** Limit of detection studies for RNA virus

Conviral copies/µL of Enterovirus mock sample	mNGS detection Normalized reads to MS2	
	With bead beating and RNaseZap (reads)	Without bead beating and RNaseZap (reads)
10^5^	160	65,138
10^4^	0	19,202
10^3^	0	774
10^2^	0	73
10	0	101

These changes increased the sensitivity of the LOD to 10 viral copies/µL (Table 5). To determine if this change was relevant to clinical samples, two samples that were either “Not Detected” for Enterovirus or had a very low number of viral reads detected with the initial protocol and had sufficient sample remaining for reanalysis were retested with the updated method. The newer protocol was able to detect Enterovirus in these two samples, where the previous method was unable to detect the virus (Table 6). These data suggested that to improve the detection of RNA organisms in the mNGS assay, this change had to be incorporated into the protocol.

**TABLE 6:** Adjusted discordant analysis for RNA virus samples^*a*^

Sample ID	Original PCR/culture result	mNGS result		Detected or not detected	Match (Y/N)
		mNGS ID	Normalized reads to MS2		
MCSF035-Run 1	Enterovirus	Enterovirus	89 reads	Detected	Y
MCSF035-Run 2	Enterovirus	Enterovirus	1,066 reads	Detected	Y
MCSF067-Run 1	Enterovirus	Negative	–	Not detected	N
MCSF067-Run 2	Enterovirus	Enterovirus	69 reads	Detected	Y

^*a*^ “–” indicates that no organism was detected in the corresponding category.

### Establishment of controls

A key element to consider with mNGS testing is its ability to determine the quality of the entire test protocol with the establishment of proper controls. This is especially important when developing mNGS into a clinical test. For this mNGS assay on CSF, two types of controls were established: external controls (both DNA and RNA) to be run in parallel with the clinical samples and spiked internal controls (both DNA and RNA). For the positive external control, two commercial standards were combined into one sample and run through the entire wet-lab and bioinformatics protocols in tandem with the clinical and contrived samples being tested (Table 7). The first community standard was the ZymoBIOMICS Microbial Community DNA Standard, and it represented the DNA fraction of bacterial and fungi targets (Table 7). All the documented organisms in the Zymo community were detected. There was a range of 2%–4% difference in the detection of bacterial genera and a 0.5% difference in the detection of fungal genera. In addition, there was a difference in the detection percentages of the bacterial and fungi organisms when the external positive control was not put through the Maxwell extraction process of the protocol (Table 7, gray highlighted cells). A tighter range of 0.5%–3% difference was observed in the detection of bacterial genera and a 0.04%–0.06% difference in the detection of fungal genera.

**TABLE 7:** External positive control results between runs^*c*^

	gDNA abundance (% estimated)	DNA external control runs (%)												
		Run 1	Run 2	Run 3	Run 4	Run 5	Run 6	Run 7-1	Run 7-2^*a*^	Run 7-3^*a*^	Run 7-4^*a*^	Run 8	Run 9-1	Run 9-2
*Pseudomonas*	12	11.98	12.00	21.62	20.57	22.53	11.44	8.91	21.44	8.35	23.54	10.19	19.90	19.46
*Escherichia^b^*	12	19.14	18.33	31.32	30.58	30.36	17.82	16.11	30.93	15.79	28.78	16.30	27.90	28.61
*Salmonella*	12	17.40	16.55	29.01	28.33	29.27	16.00	14.73	28.73	14.10	26.70	14.72	25.98	26.83
*Lactobacillus*	12	9.02	8.58	2.34	2.66	1.76	9.80	11.45	2.06	12.60	2.37	8.60	3.25	2.79
*Enterococcus*	12	8.26	10.04	1.22	1.52	1.14	10.09	11.24	1.33	11.08	1.68	11.80	3.37	2.23
*Staphylococcus*	12	8.04	6.47	0.38	0.49	0.32	6.65	7.54	0.32	8.41	0.54	7.58	0.69	0.64
*Listeria*	12	13.08	12.60	1.12	1.43	1.12	12.61	14.26	1.12	14.51	1.58	13.81	2.25	2.01
*Bacillus*	12	13.01	15.01	12.91	14.32	13.43	14.90	15.27	13.87	14.74	14.66	15.71	16.33	17.20
*Saccharomyces*	2	0.03	0.31	0.04	0.05	0.04	0.57	0.30	0.05	0.27	0.08	1.06	0.16	0.10
*Cryptococcus*	2	0.03	0.10	0.03	0.03	0.03	0.13	0.18	0.04	0.16	0.09	0.22	0.14	0.13

	gDNA/RNA abundance (% estimated)	RNA external control runs (%)							
		Run 1-1	Run 1-2	Run 1-3	Run 2	Run 3-1	Run 3-2	Run 4	Run 5
Respiratory syncytial virus (RSV)	16.7	48.15	33.75	33.77	29.71	22.11	21.67	32.91	26.99
Influenza B virus	16.7	26.29	34.89	40.58	25.60	18.08	35.49	32.47	22.72
Mammalian orthoreovirus 3	16.7	17.43	15.94	14.29	22.47	21.31	21.66	19.41	18.92
Zika virus	16.7	8.12	15.41	11.36	22.22	38.50	21.18	15.21	31.38

^*a*^ Run performed by different technicians.

^*b*^ *Escherichia* levels determined by subtracting the *Salmonella* reads from the total Enterobacteriaceae.

^*c*^ Gray highlighted cells are external controls that were not put through the extraction on the Maxwell.

The second community standard was the ATCC Virome Nucleic Acid Mix, and it represented the RNA fraction of viral targets (Table 7). All the documented organisms in the ATCC Virome Nucleic Acid Mix were detected. There was a range in the difference in the detection of each of the viral targets, but it was consistent, within 1%–2%.

An external negative control was also run in tandem with samples to determine systemic contamination due to handling or DNA/RNA already present in commercial reagents. This external negative control revealed the top organisms detected in the “kitome” (Table S2).

Overall, based on these data, the criteria for rejection of an analytical run would be as follows: (i) no detection of any of the genera listed in the DNA and RNA external positive controls and (ii) detection of other genera not listed. The actual percentage of the organism detected would not matter; however, when analyzing the percentages of each organism, one could determine if there was a lapse in the wet-lab protocol, for the external controls. For example, certain external positive controls were not run through the extraction portion of the protocol and this influenced the percentages observed (Table 7, gray highlighted cells). This suggested that one can also look at the varying percentages to see if the wet-lab preparation was performed properly and the positive controls run fully in tandem.

In addition to the external controls, internal controls were used in each sample to help determine the integrity of the sample throughout the entire protocol. The two internal control phages are the MS2 phage for monitoring RNA processing and the Lambda phage for monitoring DNA processing. Both phages were detected in every sample processed for this validation. Based on the detection of each phage in the samples tested in the validation, a threshold of detection was set to determine the integrity of the sample. The required threshold of reads for the detection of both phages was a level lower than the external water negative control. This threshold was set because the internal controls were disproportionately sequenced in samples that lacked substantial amounts of other DNA.

## DISCUSSION

Here, we present a bioinformatics pipeline that uses a category-based reporting algorithm that is meant to decrease the inherent subjectivity that comes with interpreting data from metagenomic sequencing. The assay that incorporated this bioinformatics pipeline was designed to be performed on CSF samples to aid in the diagnosis of CNS infection. The validation of this assay and pipeline revealed an overall concordance of 91.8%, with a sensitivity of 100% and a specificity of 72.4%. These test metrics differed from other mNGS assays on CSF, which have an average sensitivity of 85% and an average specificity of 99% (7–10, 23). The specificity of this mNGS assay was not as high as other marketed CSF mNGS assays, but this was strongly influenced by the initial inability to detect RNA viruses (Table 3), and improvements in our wet-lab protocol for the detection of RNA viruses will substantially improve the assay specificity.

Beyond the technical contributors to the poor detection of RNA viruses, the clinical implications of false-positive results with an mNGS assay on CSF warrant careful consideration. False-positive detections are an inherent challenge to mNGS assays because they are highly sensitive, and in low-bio-mass samples like CSF, background contamination can contribute to low-level signal (13, 14, 24). This risk can be mitigated with our category-based reporting algorithm, where lower-confidence organisms are clearly delineated and interpreted within a structured consultative framework.

There are a few large reference laboratories that offer mNGS testing on CSF, and these laboratories have tailored their bioinformatics pipelines to be more clinically focused (7, 8, 10, 23, 25, 26). All the mNGS tests clinically offered for CSF utilize different bioinformatics pipelines and, ultimately, do a good job of detecting a clinically relevant pathogen. Yet, there is still the analytical challenge of being able to confidently distinguish the clinically relevant organisms from background contamination. Furthermore, the reliability of annotated NCBI genomes could contribute to inaccuracies in mNGS testing. The category-based reporting algorithm in our bioinformatics pipeline seeks to address these issues. The designation of detected organisms into this tiered category system in a generated report allows for the user to have a list of the top organisms detected, while also reporting the other potential organisms that may be in lower abundance but that may have been missed without having an automated category-based reporting structure. Another feature built into the bioinformatics pipeline is the ability to flag background organisms commonly detected in the external negative controls and previously sequenced non-infectious CSF. As more samples are sequenced and their backgrounds routinely incorporated, this feature of utilizing previously sequenced data will continue to improve, strengthening the ability of the bioinformatics pipeline to distinguish between true signal and background noise. The historical data generated were within a single laboratory, and this has not been tested across institutions or sequencing platforms. This framework has proven effective internally, but backgrounds may vary in other settings, and a multicenter study would need to be done to confirm its applicability in other laboratories.

Our results demonstrated that most of the organisms in the positive CSF samples were detected in either Category 1 or Category 2 (Table 3). This category-based reporting still lists all organisms that were detected in the sample; yet, the generated report helps the provider distil and interpret the information. In addition, there were several positive CSF samples where our mNGS assay was able to detect additional clinically relevant organisms (Table 4). The detection of additional clinically relevant organisms in our method is consistent with prior reports describing broad detection ability with mNGS in CSF (7, 8, 10).

During our initial validation, the detection of RNA viruses was poor (Tables 5 and 6) and necessitated improvement, as many notable RNA viruses, such as arboviruses and non-polio enteroviruses, are known to cause CNS infections (1). The detection of RNA viruses in CSF has been a challenge with mNGS test development, and several groups have worked to find ways to improve the detection of RNA viruses, including viral enrichment with hybrid capture or select primer enrichment, and host depletion (27–29). Our work showed that the detection of RNA viruses is improved by splitting the CSF sample into two and performing a less harsh pre-extraction protocol for the RNA sample (Fig. 1; Table 5). This offers a more cost-effective solution to improve the detection of RNA viruses. Similarly, the higher limits of detection observed for fungal organisms may in part reflect the host DNA depletion strategy used in this workflow, as depletion of methylated eukaryotic DNA may inadvertently reduce fungal DNA.

Although many experiments were performed to optimize the detection of RNA viruses, there was a lack of breadth in the diversity of RNA viruses that were a part of the initial validation set. More work could be done to create additional contrived samples of different, clinically important RNA viruses to verify our ability to detect them; however, that would require establishing a high-biocontainment facility. The validation also excludes another major class of pathogens: parasitic organisms. Both these classes of pathogens (RNA viruses and parasites) tend to lack rapid conventional diagnostic testing methods and represent areas where mNGS may offer complementary diagnostic information. To perform a full analytical validation that encompasses a comprehensive representation of pathogens in these two groups, a multicenter study should be performed. However, the incremental improvement made in detecting RNA viruses with this mNGS assay can be of clinical value to help support providers in the clinical management of patients with RNA virus-associated CNS infections.

One key element that we have focused on developing in our mNGS assay is to ensure that proper controls are in place to substantiate the integrity of the run. This is true of any laboratory-developed test, but especially so with metagenomics, which often has a small signal-to-noise ratio. It is important to ensure accuracy and reliability throughout the entire testing process. In our validation, we encountered instances where the external and internal controls helped flag an issue in the wet-lab protocol. This allowed us to adjust and optimize the wet-lab procedure. Moving forward, any change seen in the external or internal controls would signal that there is a potential problem in the wet-lab procedure and to repeat the assay.

While the bioinformatics pipeline and tiered report enable detection of potential pathogens in the sequencing data, the data output should not be taken as fact for the diagnosis of a patient. We still advocate for this information to be used and interpreted carefully by experts within the laboratory and with assessment of the clinical information obtained from the infectious diseases and neurology teams that are evaluating and treating the patient (30). This consultative reporting model would be made of a review that incorporates the organism confidence tier, relative abundance, known pathogenicity in CSF, and potential sources of background or contamination. The finalized report is intended to function as an interpretive consultation that is intended to support careful interpretation of sequencing findings while minimizing the risk of overinterpretation.

Metagenomics sequencing as a diagnostic test in clinical infectious diseases is a rapidly growing field, but many hurdles still need to be overcome before it can be considered a gold standard in infectious disease diagnostics. This bioinformatics pipeline with a category-based reporting algorithm represents an approach to addressing known interpretive challenges with metagenomic sequencing. This study was designed as an analytical and clinical validation and did not address clinical outcomes, changes in management, or patient-level impact. Operational factors such as cost, turnaround time, and scalability were not assessed in this validation study and will require further studies. The findings with this reporting algorithm establish a validated, internally reproducible framework for CSF metagenomic sequencing that helps to advance the interpretability of metagenomic sequencing in infectious diseases diagnostics.

## Data Availability

Sequence data generated in this study have been deposited in the NCBI Sequence Read Archive (SRA) under BioProject accession PRJNA1458854. Deposited files include the pre-processed, host-filtered FASTQ files used for downstream analyses in this manuscript. Individual SRA accession numbers and associated sample metadata are provided in Table S2. Bioinformatics workflows and analysis scripts are available via GitHub (https://github.com/UofM-ARDL/csf-metagenomics) and Zenodo (https://zenodo.org/records/17148182).

## SUPPLEMENTAL MATERIAL

Supplemental material spectrum.03666-25-s0001.docx Tables S1 and S2; Fig. S1 and S2.

Table S3 spectrum.03666-25-s0002.xlsx Corresponding FastQ accession.

ASM does not own the copyrights to Supplemental Material that may be linked to, or accessed through, an article. The authors have granted ASM a non-exclusive, world-wide license to publish the Supplemental Material files. Please contact the corresponding author directly for reuse.
