# Supplementary material for: Validation of an integrated metagenomic pipeline combining optimized wet-lab processing and tiered reporting for CSF pathogen detection
Source: Microbiol Spectr. 2026 Jun 29;14(8):e03666-25. doi: 10.1128/spectrum.03666-25 (PMC13436388; doi:10.1128/spectrum.03666-25)
Supplement: Supplementary file 1 [file spectrum.03666-25-s0001.docx]

**SUPPLEMENTAL MATERIAL**

***Detailed Summary of Validation Samples***

Below in **Supplementary Table S1** is a summary of all the clinical and contrived samples tested in this validation minus the limit of detection (LOD) studies (described in detail later in supplemental data). The blue and orange highlighted cells are the discordant results, which are broken down and investigated in an adjusted concordance analysis in the next section.

**Supplementary Table S1-Detailed Summary of Validation Set**

| **Sample ID** | **Original PCR/**  **Culture Result** | **mNGS Result** | | **Detected or Not Detected** | **Match**  **(Y/N)** |
| --- | --- | --- | --- | --- | --- |
|  |  | **mNGS ID** | **Normalized RPKR to Lambda (* Normalized Reads to MS2)** |  |  |
| MCSF004 | *Streptococcus pneumoniae* | *Streptococcus pneumoniae* | 0.841 | Detected but under threshold | N |
| MCSF006 | *Streptococcus pneumoniae* | *Streptococcus pneumoniae* | 403.988 | Detected | Y |
| MCSF026 | *Streptococcus pneumoniae* | *Streptococcus pneumoniae* | 0.597 | Detected but under threshold | N |
| MCSF039 | *Streptococcus pneumoniae* | *Streptococcus pneumoniae* | 2.0130 | Detected | Y |
| MCSF063-Run 1 | *Streptococcus pneumoniae* | *Streptococcus pneumoniae* | 10.4596 | Detected | Y |
| MCSF063-Run 2 | *Streptococcus pneumoniae* | *Streptococcus pneumoniae* | 2.8726 | Detected | Y |
| MCSF063-Run 3 | *Streptococcus pneumoniae* | *Streptococcus pneumoniae* | 22 | Detected | Y |
| MCSF063-Run 3.1 | *Streptococcus pneumoniae* | *Streptococcus pneumoniae* | 18 | Detected | Y |
| MCSF066-Run 1 | *Streptococcus pneumoniae* | *Streptococcus pneumoniae* | 249.0242 | Detected | Y |
| MCSF066-Run 2 | *Streptococcus pneumoniae* | *Streptococcus pneumoniae* | 262 | Detected | Y |
| MCSF066-Run 2.1 | *Streptococcus pneumoniae* | *Streptococcus pneumoniae* | 262 | Detected | Y |
| MCSF077 | *Cutibacterium acnes* | *Cutibacterium acnes* | 0.2074 | Detected but under threshold | N |
| Study #85B | *Enterococcus faecium* | *Enterococcus faecium* | 0.4183 | Detected | Y |
| *Listeria monocytogenes-*Straight Mock | *Listeria monocytogenes* | *Listeria monocytogenes* | 690.5340 | Detected | Y |
| *Cutibacterium acnes-*Straight Mock | *Cutibacterium acnes* | *Cutibacterium acnes* | 809.6988 | Detected | Y |
| *Streptococcus agalactiae* LOD 10^6^ | *Streptococcus agalactiae* | *Streptococcus agalactiae* | 14.65202 | Detected | Y |
| MCSF078 | *Neisseria meningitidis* | *Neisseria meningitidis* | 278.0409 | Detected | Y |
| Study #67B | *Escherichia coli* | *Escherichia coli* | 24.6284 | Detected | Y |
| Study #67C | *Escherichia coli* | *Escherichia coli* | 92.9762 | Detected | Y |
| Study #86 | *Klebsiella pneumoniae* | *Klebsiella variicola* | 0.2565 | Detected^ | Y |
| *Neisseria meningitidis-*Straight Mock-1 | *Neisseria meningitidis* | *Neisseria meningitidis* | 430.2015 | Detected | Y |
| *Escherichia coli-*  Straight Mock-1 | *Escherichia coli* | *Escherichia coli* | 225.5459 | Detected | Y |
| *Escherichia coli-*  Straight Mock-2 | *Escherichia coli* | *Escherichia coli* | 222.2381 | Detected | Y |
| *Neisseria meningitidis-*Straight Mock-2 | *Neisseria meningitidis* | *Neisseria meningitidis* | 434.1923 | Detected | Y |
| *Haemophilus influenzae-*Straight Mock | *Haemophilus influenzae* | *Haemophilus influenzae* | 403.7763 | Detected | Y |
| *Haemophilus influenzae* LOD 10^6^ | *Haemophilus influenzae* | *Haemophilus influenzae* | 59.73279 | Detected | Y |
| Study #4 | *Cryptococcus neoformans* | *Cryptococcus neoformans* | 4.2287 | Detected | Y |
| Study #35 | *Cryptococcus neoformans* | Negative | *Detected in non-STD file at 0.1611 | Detected but under threshold | N |
| Study #39 | *Cryptococcus neoformans* | *Cryptococcus neoformans* | 41.9418 | Detected | Y |
| Study #94-Run 1 | *Cryptococcus neoformans* | *Cryptococcus neoformans* | 3.8195 | Detected | Y |
| Study #94-Run 1.1 | *Cryptococcus neoformans* | *Cryptococcus neoformans* | 3.9858 | Detected | Y |
| *Candida albicans*-  Straight Mock-1 | *Candida albicans* | *Candida albicans* | 390.3391 | Detected | Y |
| *Candida albicans-*  Straight Mock-2 | *Candida albicans* | *Candida albicans* | 383.6221 | Detected | Y |
| *Cryptococcus neoformans-* Straight Mock | *Cryptococcus neoformans* | *Cryptococcus neoformans* | 519.9409 | Detected | Y |
| *Cryptococcus neoformans* LOD 10^6^ | *Cryptococcus neoformans* | *Cryptococcus neoformans* | 21.14510 | Detected | Y |
| MCSF001 | Human Herpesvirus 6 | Human Herpesvirus 6 | 0.005 | Detected | Y |
| MCSF010 | Herpes Simplex Virus 1 | Herpes Simplex Virus 1 | 7.997 | Detected | Y |
| MSCF012 | Human Herpesvirus 6 | Negative | - | Not Detected | N |
| MCSF017 | Herpes Simplex Virus 2 | Herpes Simplex Virus 2 | 0.010 | Detected | Y |
| MCSF034 | Varicella Zoster Virus | Varicella Zoster Virus | 0.002 | Detected | Y |
| MCSF038-Run 1 | Human Herpesvirus 6 | Human Herpesvirus 6 | 75.9927 | Detected | Y |
| MCSF038-Run 2 | Human Herpesvirus 6 | Human Herpesvirus 6 | 27.9 | Detected | Y |
| MCSF038-Run 2.1 | Human Herpesvirus 6 | Human Herpesvirus 6 | 37 | Detected | Y |
| MCSF041 | Herpes Simplex Virus 2 | Herpes Simplex Virus 2 | 0.008 | Detected | Y |
| MCSF045 | Human Herpesvirus 6 | Human Herpesvirus 6 | 0.001 | Detected | Y |
| MCSF047 | Herpes Simplex Virus 2 | Herpes Simplex Virus 2 | 0.0015 | Detected | Y |
| MCSF048 | Herpes Simplex Virus 1 | Herpes Simplex Virus 1 | 0.100 | Detected | Y |
| MCSF049 | Human Herpesvirus 6 | Negative | - | Not Detected | N |
| MCSF051 | Herpes Simplex Virus 1 | Herpes Simplex Virus 1 | 7.8531 | Detected | Y |
| MCSF062-Run 1 | Human Herpesvirus 6 | Human Herpesvirus 6 | 0.0024 | Detected | Y |
| MCSF062-Run 2 | Human Herpesvirus 6 | Human Herpesvirus 6 | 0.0026 | Detected | Y |
| MCSF062-Run 2.1 | Human Herpesvirus 6 | Human Herpesvirus 6 | 0.0041 | Detected | Y |
| MCSF076 | Herpes Simplex Virus 1 & Cytomegalovirus | Herpes Simplex Virus 1 & Cytomegalovirus | 0.0011 (HSV-1)  7.8395 (CMV) | Detected | Y |
| MCSF075 | Human Herpesvirus 6 | Human Herpesvirus 6 | 0.0008 | Detected | Y |
| MCSF091 | Human Herpesvirus 6 | Human Herpesvirus 6 | 0.0023 | Detected | Y |
| MCSF093 | Human Simplex Virus 2 | Herpes Simplex Virus 2 | 11.3015 | Detected | Y |
| MCSF095 | Varicella Zoster Virus | Varicella Zoster Virus | 0.1258 | Detected | Y |
| MCSF100 | Herpes Simplex Virus 2 | Herpes Simplex Virus 2 | 0.0939 | Detected | Y |
| MCSF101 | Human Herpesvirus 6 | Human Herpesvirus 6 | 0.0009 | Detected | Y |
| MCSF011 | Enterovirus | Negative | - | Not Detected | N |
| MCSF027 | Enterovirus | Negative | - | Not Detected | N |
| MCSF029 | Enterovirus | Enterovirus | 115 reads* | Detected | Y |
| MCSF030 | Enterovirus | Enterovirus | 4135 reads* | Detected | Y |
| MCSF035-Run 1 | Enterovirus | Enterovirus B | 89 reads* | Detected | Y |
| MCSF035-Run 2 | Enterovirus | Enterovirus | 1066 reads* | Detected | Y |
| MCSF064 | Enterovirus | Enterovirus B | 18 reads* | Detected | Y |
| MCSF065 | Enterovirus | Enterovirus | 5 reads* | Detected | Y |
| MCSF067-Run 1 | Enterovirus | Negative | - | Not Detected | N |
| MCSF067-Run 2 | Enterovirus | Enterovirus | 69 reads* | Detected | Y |
| MCSF068 | Parechovirus | Negative | - | Not Detected | N |
| MCSF096 | Enterovirus | Negative | - | Not Detected | N |
| MCSF097 | Enterovirus | Enterovirus B | 4 reads* | Detected | Y |
| MCSF102 | Enterovirus | Enterovirus B | 581 reads* | Detected | Y |
| Study #18 | Enterovirus | Enterovirus B | 18 reads* | Detected | Y |
| Study #44-Run 1 | Enterovirus | Enterovirus B | 1709 reads* | Detected | Y |
| Study #44-Run 2 | Enterovirus | Enterovirus B | 1113 reads* | Detected | Y |
| Study #53 | Enterovirus | Enterovirus B | 1113 reads* | Detected | Y |
| MCSF053 | Negative | Negative | - | Not Detected | Y |
| MCSF054 | Negative | Negative | - | Not Detected | Y |
| MCSF055 | Negative | Negative | - | Not Detected | Y |
| MCSF056 | Negative | Negative | - | Not Detected | Y |
| MCSF057 | Negative | Negative | - | Not Detected | Y |
| MCSF069 | Negative | Negative | - | Not Detected | Y |
| MCSF070 | Negative | Negative | - | Not Detected | Y |
| MCSF071 | Negative | Negative | - | Not Detected | Y |
| MCSF072 | Negative | Negative | - | Not Detected | Y |
| MCSF073 | Negative | Negative | - | Not Detected | Y |
| MCSF074 | Negative | Negative | - | Not Detected | Y |
| MCSF079 | Negative | Negative | - | Not Detected | Y |
| MCSF080 | Negative | Negative | - | Not Detected | Y |
| MCSF081 | Negative | Negative | - | Not Detected | Y |
| MCSF082 | Negative | Negative | - | Not Detected | Y |
| MCSF083 | Negative | Negative | - | Not Detected | Y |
| MCSF084 | Negative | *Methylorubrum rhodinum* | 0.1565 | Detected Something Significant | N |
| MCSF087 | Negative | Negative | - | Not Detected | Y |
| MCSF088 | Negative | Negative | - | Not Detected | Y |
| MCSF089 | Negative | Negative | - | Not Detected | Y |
| MCSF090 | Negative | Negative | - | Not Detected | Y |

Orange highlighted cells are the discordant results that remained discordant. Blue highlighted cells are results that were initially discordant but changed to concordant upon review (described in *Adjusted Concordance Analysis* section of Supplementary material.

^*K. variicola* is part of the *K. pneumoniae* group

***Adjusted Concordance Analysis***

There were four samples in the discordant results in which the organism was detected but below the set threshold (2-*S. pneumoniae*, 1 *C. neoformans*, and 1 *C. acnes*) (**Supplementary Table S1**). In the reporting of results, we plan to have a mNGS team review the results and discuss what is reported in the patient’s chart. In these four instances, the organism under threshold would be reported out with a disclaimer, “The following targets were detected, but under the set thresholds so interpret with caution.” With this modification, the status of three of the four discordant results can be considered concordant. The one case of *C. neoformans* was not in the standardized file output, which is the file that will be used to determine what is and what is not reported. Therefore, it was kept as a discordant result.

There was one sample where the genus (*Klebsiella*) mNGS result matched the original PCR genus; however, there was a difference in the call of the bacterial species (**Supplementary Table S1, Study #86**). The original PCR result was *Klebsiella pneumoniae*, while the mNGS result was *Klebsiella variicola*. It is known that *K. variicola* is not part of the M/E PCR panel, but it is part of the *K. pneumoniae complex*^1^. This would be considered a significant result that would be reported as *Klebsiella species*. Therefore, the result was changed to be concordant with the original result.

There was one sample that was negative by the conventional methods, but mNGS detected the organism *Methylorubrum rhodinum*. Upon review, this organism is not a significant result, as it is not known to be a human pathogen, and is typically found in soil^2^. This result was changed to a concordant result of “Not Detected” as this would not be reported in the patient’s chart upon review with the mNGS team.

There are two viral DNA samples where Human Herpesvirus 6 (HHV-6) was positive with the original M/E panel PCR, but not detected with our mNGS assay. Our DNA virus LOD studies resulted in an LOD of 10 viral copies/µL. It is possible that the virus was present in low amounts initially and upon the freeze/thaw cycle of the specimen that the virus was lost. It must also be noted that the detection of HHV-6 with the M/E panel PCR is known to have a lower specificity and generally requires a confirmation of the result as false positives are known to occur^3^. Both these patients did not have confirmatory testing for HHV-6 with other methods; therefore, these samples may be false positives of HHV-6 on the M/E panel PCR, and it was not detected it with our mNGS assay because the samples are not truly positive for HHV-6. Since there were no additional confirmatory tests to perform at the time of analysis, these results will remain discordant.

There were five viral RNA samples that were detected with the original M/E panel PCR but then subsequently not detected with our mNGS assay. This is not too surprising given RNA stability. A re-run of the sample for which additional CSF was available (MCSF067) was performed to see if the Enterovirus could be detected with the less harsh extraction method (**Supplementary Table S1**). These data suggested that the RNA was being destroyed at some point in the wet lab protocol with the initial samples. Therefore, this sample, shown in **Supplementary Table S1,** was changed to concordant since the re-run results showed detection of Enterovirus in the sample. The following four viral RNA samples (MCSF011, MCSF027, MCSF068 and MCSF096) were unable to be repeated, as no additional CSF sample remained, so these samples remained discordant. However, a repeat was performed on a sample that had initial low Enterovirus detection with only 89 reads (MCSF035, **Supplementary Table S1**). The re-run of MCSF035 with the less harsh extraction pipeline showed an increase in the number of reads mapping to Enterovirus further suggesting the systematic detection of Enterovirus across samples (**Table 3**).

***External Negative Controls***

Our negative water external controls are a representation of the “kitome” that we see from all the reagents in our wet lab protocol ^4–8^. Below is a table that depicts the top organisms that were detected within the negative external water controls (**Supplementary Table S2)**.

**Supplementary Table S2- Top Organisms Detected in Negative External Water Controls**

| **DNA Organisms** | **Number of Experiments** | **Average Reads**  **per Experiment** | **Average Normalized**  **RPKR to Lambda** |
| --- | --- | --- | --- |
| *Burkholderia* | 13 | 5,207,622 | 643.27 |
| *Leifsonia* | 13 | 85,703 | 8.45 |
| *Sarumanvirus* | 13 | 39,037 | 4.71 |
| *Cutibacterium* | 13 | 15,961 | 12.58 |
| *Microbacterium* | 13 | 6,915 | 0.92 |
| *Streptomyces* | 13 | 6,793 | 1.07 |
| *Pseudomonas* | 13 | 4,268 | 2.60 |
| *Bradyrhizobium* | 13 | 3,541 | 0.46 |
| *Stutzerimonas* | 13 | 2,500 | 1.89 |
| *Paraburholderia* | 13 | 2,093 | 0.30 |
| *Corynebacterium* | 13 | 1,820 | 1.54 |
| *Nocardioides* | 13 | 1,652 | 0.28 |
| *Synechococcus* | 13 | 1,435 | 0.17 |
| *Staphylococcus* | 13 | 1,324 | 5.98 |
| *Curtobacterium* | 13 | 1,265 | 0.17 |
| *Sphingomonas* | 13 | 1,241 | 0.29 |
| *Rhodococcus* | 13 | 1,104 | 0.25 |
| *Agromyces* | 13 | 1,082 | 0.14 |
| *Sphingobium* | 13 | 1,078 | 0.27 |
| *Cryobacterium* | 13 | 1,077 | 0.15 |
| **RNA Organisms** | **Number of Experiments** | **Average Reads per Experiment** | |
| Murine leukemia virus | 7 | 8 | |
| Moloney murine leukemia virus | 6 | 12 | |
| Tobacco vein clearing virus | 4 | 1 | |
| Diplodia scrobiculata RNA virus 1 | 3 | 3 | |
| Koala retrovirus | 3 | 3 | |
| Human immunodeficiency virus 1 | 2 | 333 | |
| Woodchuck hepatitis virus | 2 | 77 | |
| Gammaretrovirus | 2 | 6 | |
| Orthohepadnavirus | 2 | 6 | |
| Enterovirus B | 2 | 3 | |
| Friend murine leukemia virus | 2 | 2 | |
| Harvey murine sarcoma virus | 2 | 2 | |
| Enterobacteria phage GA | 2 | 1 | |

**Supplementary Figure S1 - Example of a Kraken2 report (Sample MCSF093)**

Total reads: 19,514,479

Useable reads: 16,066,227

Human reads: 15,853,126

Human mRNA reads: 0

Lambda phage spike-in reads: 8,292

MS2 phage spike-in reads: 0

Unique filtered reads: 191,036

Organisms with RPKM(reads per thousand filtered reads) > 5σ:

Organisms with unknown RPKM distributions (observed in n 'negative' datasets), but are above species-specific thresholds listed below, reads per thousand filtered reads:

Human alphaherpesvirus 1: 0.0157, n=2

Human alphaherpesvirus 2: 11.3015, n=2

All organisms above kingdom threshold, reads per thousand filtered reads:

Homo sapiens: 45.4365

Toxoplasma gondii: 30.5440

Acinetobacter baumannii: 5.2713

Staphylococcus aureus: 1.9054

Mycobacterium leprae: 1.7902

Burkholderia cenocepacia: 1.4343

Klebsiella pneumoniae: 1.2511

Vibrio parahaemolyticus: 1.0522

Panine alphaherpesvirus 3: 0.0209

Thresholds:

Species thresholds, reads per thousand filtered reads:

Cryptococcus neoformans: 2

Enterococcus faecium: 1.0

Escherichia coli: 3

Human alphaherpesvirus 1: 0.0

Human alphaherpesvirus 2: 0.0

Human alphaherpesvirus 3: 0.0

Human betaherpesvirus 6B: 0.0

Klebsiella: 1.5

Klebsiella pneumoniae: 1.5

Pseudomonas aeruginosa: 1

Staphylococcus aureus: 3

Streptococcus pneumoniae: 1

Streptomyces: 3.5

betaherpesvirus 6: 0.0

gammaherpesvirus 4: 0.0

Kingdom thresholds, reads:

Archaea: 10

Bacteria: 200

Eukaryota: 100

Viruses: 3

**Supplementary Figure S2 - Example of a Kaiju report (Sample MCSF065)**

Total reads: 20,629,403

Useable reads: 17,824,465

Human reads: 8,765,763

Human mRNA reads: 0

Lambda phage spike-in reads: 1,412

MS2 phage spike-in reads: 150

Unique filtered reads: 5,600,919

Organisms not seen in input, reads:

Enterovirus: 5

Coxsackievirus B3: 2

Enterovirus B: 2

Squirrel monkey retrovirus: 1

Gammaretrovirus: 1

Harvey murine sarcoma virus: 1

Citrus endogenous pararetrovirus: 1

Gibbon ape leukemia virus: 1

Organisms also seen in input, reads:

Tobacco vein clearing virus: 8

Diplodia scrobiculata RNA virus 1:

**Supplemental Table S3-Detailed Summary of Validation Samples and NCBI Reference FastQ Accession**

Legend: *Water Control library failed and unable to sequence

**SUPPLEMENTAL REFERENCES**

1.	Rodríguez-Medina, N., Barrios-Camacho, H., Duran-Bedolla, J. & Garza-Ramos, U. Klebsiella variicola: an emerging pathogen in humans. *Emerg. Microbes Infect.* **8**, 973–988 (2019).

2.	Green, P. N. & Ardley, J. K. Review of the genus Methylobacterium and closely related organisms: a proposal that some Methylobacterium species be reclassified into a new genus, Methylorubrum gen. nov. *Int. J. Syst. Evol. Microbiol.* **68**, 2727–2748 (2018).

3.	Leber, A. L. *et al.* Multicenter Evaluation of BioFire FilmArray Meningitis/Encephalitis Panel for Detection of Bacteria, Viruses, and Yeast in Cerebrospinal Fluid Specimens. *J. Clin. Microbiol.* **54**, 2251–2261 (2016).

4.	Laurence, M., Hatzis, C. & Brash, D. E. Common contaminants in next-generation sequencing that hinder discovery of low-abundance microbes. *PloS One* **9**, e97876 (2014).

5.	Simner, P. J. & Salzberg, S. L. The Human ‘Contaminome’ and Understanding Infectious Disease. *N. Engl. J. Med.* **387**, 943–946 (2022).

6.	Eisenhofer, R. *et al.* Contamination in Low Microbial Biomass Microbiome Studies: Issues and Recommendations. *Trends Microbiol.* **27**, 105–117 (2019).7.	Salter, S. J. *et al.* Reagent and laboratory contamination can critically impact sequence-based microbiome analyses. *BMC Biol.* **12**, 87 (2014).

8.	Chrisman, B. *et al.* The human ‘contaminome’: bacterial, viral, and computational contamination in whole genome sequences from 1000 families. *Sci. Rep.* **12**, 9863 (2022).
